# Identification of a Bitter-Taste Receptor Gene Repertoire in Different Lagomorphs Species

**DOI:** 10.3389/fgene.2016.00055

**Published:** 2016-04-06

**Authors:** Ana M. Ferreira, Andreia T. Marques, Luca Fontanesi, Carl-Gustaf Thulin, Elvira Sales-Baptista, Susana S. Araújo, André M. Almeida

**Affiliations:** ^1^Instituto de Ciências Agrárias e Ambientais Mediterrânicas, Universidade de ÉvoraÉvora, Portugal; ^2^Laboratório de Biotecnologia de Células Vegetais, Instituto de Tecnologia Química e Biológica António Xavier, Universidade Nova de LisboaOeiras, Portugal; ^3^Dipartimento di Scienze Veterinarie e Sanità Pubblica, Università degli Studi di MilanoMilan, Italy; ^4^Department of Agricultural and Food Sciences, Division of Animal Sciences, University of BolognaBologna, Italy; ^5^Department of Wildlife, Fish and Environmental Studies, Swedish University of Agricultural SciencesUmeå, Sweden; ^6^Departamento de Zootecnia, Universidade de ÉvoraÉvora, Portugal; ^7^Plant Biotechnology Laboratory, Department of Biology and Biotechnology “L. Spallanzani", Università degli Studi di PaviaPavia, Italy; ^8^Ross University School of Veterinary Medicine, BasseterreSaint Kitts and Nevis

**Keywords:** bitter-taste receptors, TAS2R, Lagomorph, *Lepus*, *Oryctolagus*, *Romerolagus*, *Sylvilagus*

## Abstract

The repertoires of bitter-taste receptor (T2R) gene have been described for several animal species, but these data are still scarce for Lagomorphs. The aim of the present work is to identify potential repertoires of T2R in several Lagomorph species, covering a wide geographical distribution. We studied these genes in *Lepus timidus*, *L. europaeus, Oryctolagus cuniculus algirus, Romerolagus diazi*, and *Sylvilagus floridanus*, using *O. cuniculus cuniculus* as control species for PCR and DNA sequencing. We studied the identities of the DNA sequences and built the corresponding phylogenetic tree. Sequencing was successful for both subspecies of *O. cuniculus* for all T2R genes studied, for five genes in *Lepus*, and for three genes in *R. diazi* and *S. floridanus.* We describe for the first time the partial repertoires of T2R genes for Lagomorphs species, other than the common rabbit. Our phylogenetic analyses indicate that sequence proximity levels follow the established taxonomic classification.

## Introduction

Bitter taste is associated with sensitivity to toxins and food choices in mammals. At the molecular level, its signaling occurs via bitter G-protein-coupled taste receptors (T2R). Taste receptors were first identified on the tongue; however, recent studies have shown that taste receptors are also expressed in several other tissues, such as gastrointestinal epithelia or respiratory tract (Lee and Cohen, 2014; Vegezzi et al., 2014). The repertoire of T2R genes has been described for several animal species, being nearly fully characterized for humans and mice (Behrens and Meyerhof, 2009, 2011), partially for sheep (Ferreira et al., 2013) and a variety of wild and domestic ruminants (Ferreira et al., 2015). Information on T2R sequences in Lagomorphs is currently only available for the common rabbit (*Oryctolagus cuniculus*) and one pika species (*Ochotona princeps*), based on the annotation of their sequenced genomes, OryCun2.0 and OchPri2.0, respectively (Lindblad-Toh et al., 2011; Carneiro et al., 2014; Flicek et al., 2014).

The Lagomorpha is a globally distributed genus with relatively few species. In this study we focus on the following species and subspecies: Domestic European rabbit (*O. cuniculus cuniculus*), Wild Iberian rabbit (*O. cuniculus algirus*), Brown hare (*Lepus europaeus*), Mountain hare (*Lepus timidus*), from Europe, the Volcano rabbit (*Romerolagus diazi*) from the Mexico, the Eastern cottontail (*Sylvilagus floridanus*) and the American pika (*O. princeps*), both North American species. These species have different feeding habits ranging from grasses, leaves, buds, tree bark, and roots for the rabbit species (Tislerics, 2000; Miller et al., 2014), grasses, herbs, and crops during summer and twigs, buds, shrub bark, small trees, and young fruit tree bark during winter for the hare species and the cottontail (Detweiler, 2000; Vu, 2001), leaves of grasses and some spiny herbs for the Volcano rabbit (Fa and Bell, 1990) and green plants like grasses, sedges, thistles, and fireweed for Pika.

Considering all these different nutritional choices of these species of lagomorphs, the aim of this study is to identify the potential repertoires of T2R among them and establish an overview of T2R sequence variation over this wide geographical areas, species, and different dietary preferences. To the best of our knowledge, it is the first time that this subject is addressed in lagomorph species with such a broad range of geographical distribution and dietary preferences and choices. This work will therefore shed light on bitter taste perception in non-model lagomorph species.

## Materials and Methods

DNA samples were extracted from blood, tissue or dropping of three specimens of each Lagomorph species, using the Qiagen Animal DNeasy Blood and Tissue Kit (QIAGEN, Venlo, the Netherlands). The only exception was the volcano rabbit (*R. diazi*), for which it was only possible to obtain one specimen. Domestic European rabbit (*O. cuniculus cuniculus*) and Wild Iberian rabbit (*O. cuniculus algirus*) samples were obtained from the Veterinary Faculty of the University of Lisbon, Portugal (Almeida et al., 2010). Brown hare (*L. europaeus*) and Mountain hare (*L. timidus*) samples were obtained from Sweden, whilst Volcano rabbit (*R. diazi*) and Eastern cottontail (*S. floridanus*) samples were obtained, respectively, from Mexico and from the United States.

The coding DNA sequences of the seven T2R genes (T2R1, T2R3, T2R4, T2R7, T2R9, T2R16, and T2R41) previously described for common rabbit (*O. cuniculus*) were retrieved from Ensembl database (EnsEMBL release 72 – June 2013) and used to design PCR primers in Primer3 software^1^. T2R genes contain no introns, so the coding sequence corresponds to the only exon. Primers sequences used and expected fragment sizes are presented in **Table 1**.

**Table 1:** PCR results of the T2R genes in the six animal species and seven genes analyzed.

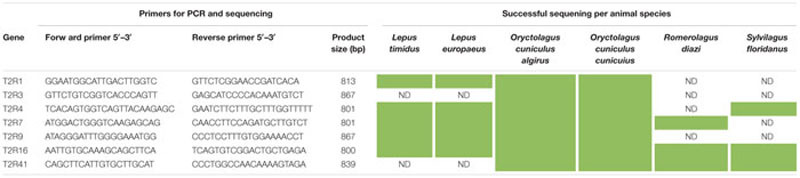

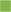
: successful sequencing reaction results, ND: failed amplification/sequening.

PCR reactions were carried out in a Bio-Rad C1000 Thermal Cycler (Bio-Rad Laboratories, Munich, Germany), using standard conditions (Ferreira et al., 2013). The PCR products, consisting of a unique band with the expected size, were purified using the QIAquick PCR Purification Kit (Qiagen, Venlo, the Netherlands) and sequenced (Sanger method) as a purchased service from Stabvida (Stabvida, Caparica, Portugal).

Sequencing data was manually checked using Chromas Lite 2.1.1^2^ for visualization; FASTA files containing the DNA sequences were then used for conversion to protein sequences, using DNA to protein sequence converter at http://www.ebi.ac.uk/Tools/st/emboss_transeq/. Protein sequences in FASTA format were then used for multiple sequence alignment by multiple sequence comparison by log-expectation (MUSCLE), freely available online at http://www.ebi.ac.uk/Tools/msa/muscle (version 3.8.31; Edgar, 2004). For this MUSCLE analysis, we also included the sequences annotated in the OchPri2.0 genome version (9) for the corresponding T2R in *O. princeps* to provide an overview of all Lagomorph T2R genes available. MUSCLE was also used to establish a percent identity matrix.

The MUSCLE data [clustal format] were used for phylogenetic analysis using the software MEGA version 6 (Tamura et al., 2013), available at http://www.megasoftware.net. Maximum Likelihood method of analysis and the bootstrap value of 500 replicates were used to infer the phylogenetic tree, and genetic distances were computed using the JTT matrix-based method (Jones et al., 1992).

### Animal Experimentation and Welfare Disclaimer

The animal work herein described followed all relevant rules on animal experimentation in Portugal and the European Union. Accordingly and because in this trial did not involve animal experimentation *per se*, but instead only collected samples, no Ethics committee or IACUC (Institutional Animal Care and Use Committee) approval was necessary. In fact all the DNA were obtained from archived samples from previous studies: New Zealand White and Iberian wild rabbit species DNA were obtained from a previous experiment approved by the ethics committee of the Faculty of Veterinary Medicine of the University of Lisbon in Portugal [for further details please refer to Almeida et al. (2010)], whereas the other lagomorph DNA was extracted from droppings from wild animals.

## Results and Discussion

In this study, we obtained partial T2R genes for five additional Lagomorphs in addition to European rabbit and pika, already sequenced (Flicek et al., 2014). PCR amplifications and sequencing reactions were successful for both subspecies of *O. cuniculus* for all T2R genes sequenced. In genus *Lepus* only five T2R genes could be sequenced because of failed PCR amplification. Similarly, only three genes were amplified for *R. diazi* and *S. floridanus* (**Table 1**). Possibly genes with lower similarity levels, or with high similarities but containing gaps in the template sequence at the annealing point of the primers, could not be amplified by PCR and, therefore, were not selected for sequencing. The amplifications may have additionally failed because the sequences are so different that the primers designed for the *O. cuniculus* simply could not be amplified or alternatively because some genes are simply not present in the repertoire of the species. However, it must be stated that no genome sequences are, to the best of our knowledge, available to any of the species studied. For that reason we have chosen the use the available *O. cuniculus* primers for all species. A strategy based on degenerate primers could also be considered. Such strategy would, however, require lengthy optimized amplification procedures and higher success rates than the one we have obtained in the present study would not be guaranteed (Linhart and Shamir, 2002). For that reason, and similarly to our previous research in ruminants (Ferreira et al., 2015), we have chosen not to conduct it. Protein sequences obtained by conversion of DNA sequences are presented in **Supplementary Data Sheet 1**.

Percent identity matrix for protein sequences (**Supplementary Data Sheet 2**) shows that identities between species for each receptor successfully sequenced ranges from 81% to nearly 100%, indicating a strong conservation of these genes within the order Lagomorpha. The pika sequences were the most different, in some cases having similarities of about 70%. However, as the sequences were not obtained from Ensembl, and sequenced using other primers, different lengths of the amplicons might explain some discrepancies in the frequency similarity. Also pseudogenization could have occurred, or even the gene can have a different (closer) homolog in other species with different T2R numbering.

**FIGURE 1:**
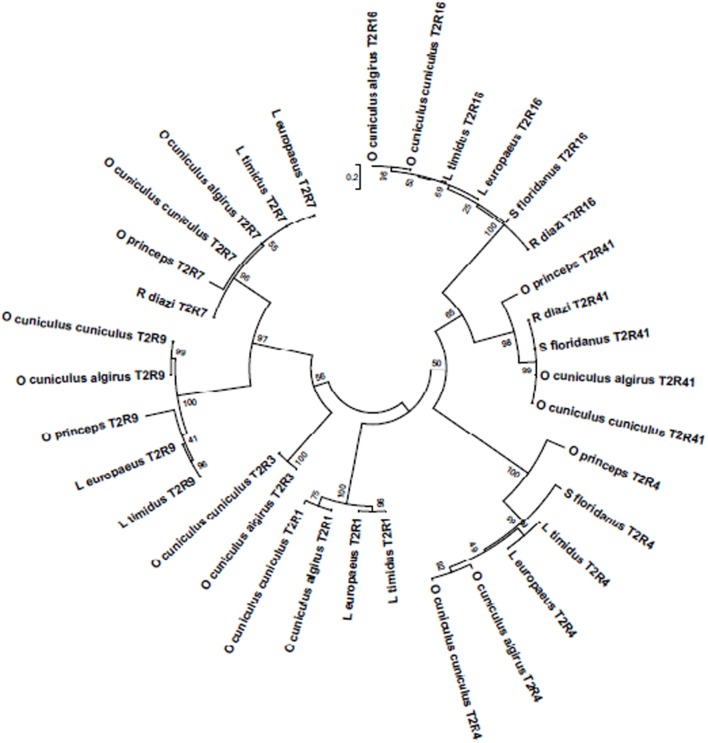
**Phylogenetic tree of the T2R amplified in the six lagomorph species studies.** Evolutionary relationships of taxa. The evolutionary history was inferred by using the Maximum Likelihood method based on the JTT matrix-based model (Jones et al., 1992). The tree with the highest log likelihood (-2253.6927) is shown. Initial tree(s) for the heuristic search were obtained automatically by applying Neighbor-Join and BioNJ algorithms to a matrix of pairwise distances estimated using a JTT model, and then selecting the topology with superior log likelihood value. The tree is drawn to scale, with branch lengths measured in the number of substitutions per site. The analysis involved 34 amino acid sequences. All positions containing gaps and missing data were eliminated. There were a total of 94 positions in the final dataset. Evolutionary analyses were conducted in MEGA6 (Tamura et al., 2013).

The phylogenetic tree of the T2R amplified in the six lagomorph species and pika sequences obtained from literature (**Figure 1**) show that the sequences are most similar between species of the same genus, despite being from separate geographic regions. We observe for the majority of T2R genes an *Oryctolagus* cluster and a *Lepus* cluster, and a separation from these two clusters from the *Romerolagus* and the *Sylvilagus* representatives. In *T2R41* however, all the sequences analyzed in the leporid species that could be obtained (*O. cuniculus*, *R. diazi*, and *S. floridanus*) are 100% identical and therefore form one unique phylogenetic cluster. This may indicate that some of the bitter receptors might be responsible for a broad detection of bitter compounds in the diet of these animals, independent of their taxonomic proximity or habitat; or that they could play other relevant functions that might have produced this evolutionary constrain. In human, the T2R41’s ligand is cloramphenicol, but it is unknown which substances are detected by this receptor in other species (Thalmann et al., 2013). The sequences from pika added is placed in a different phylogenetic branch, but in general also separate further away from the *Oryctolagus* and *Lepus* clusters. That *R. diazi* and *S. floriandus* sequences failed to amplify more often than the other species, might mean that T2R sequences for these two species have lower similarities that do not allow amplification with the used primers.

In the future, ligand studies may unravel which substances are detected by each receptor in animals, which could help understanding the relation between DNA/protein sequences and the biological relevance of each receptor in each habitat/type of diet. This study provides already a first overview of evolutionary differences of T2R genes among several species of the Order Lagomorpha.

## Author Contributions

AF, AM, LF, C-GT, and SA carried out the samples preparation and the molecular genetic studies. AF performed the phylogenetic analysis. AF, ES-B, SA, and AA conceived of the study, and participated in its design and coordination. All authors helped to draft the manuscript. All authors read and approved the final manuscript.

## Conflict of Interest Statement

The authors declare that the research was conducted in the absence of any commercial or financial relationships that could be construed as a potential conflict of interest.

## Footnotes

1 http://frodo.wi.mit.edu/

2 http://technelysium.com.au/

## Supplementary material

DATA SHEET S1 **Bitter-taste receptor (T2R) protein sequences obtained for the different species studied**.

DATA SHEET S2 **Percent identity matrix obtained by MUSCLE analysis**.
