# Supplementary material for: Identification of a Bitter-Taste Receptor Gene Repertoire in Different Lagomorphs Species
Source: Front Genet. 2016 Apr 6;7:55. doi: 10.3389/fgene.2016.00055 (PMC4822227; doi:10.3389/fgene.2016.00055)
Supplement: DATA SHEET S1 — Bitter-taste receptor (T2R) protein sequences obtained for the different species studied. [file Data_Sheet_1.DOCX]

> L_timidus_T2R1

LLSCLAVSRICLQLLLFYMNLFFLSLTQVSTFVENFVIVLFTNEVGLWLATWLGVFYCIK

ITNFHHPLFLWLKMRISQLLPWLVLGSLLCAAISSGICSMQSWMSLEKLLQTFLSKNATN

PIKEISALSSFSVIGGFPFPFFIFLVAALLLILSLGRHTWQLRSSTAGTRFPRRSAHVRA

LLSVLSFLFLYFSHFVVVLLLSSQVFPPGSLTFMLCLLISGAYPSGHSVILILGNSKLKQ

NAKRLL

> L_timidus_T2R4

LFSLGITRFLTLGLFLLNIIYHTAAGVERSVYISIFLQVCWMFLDSISVWFVTLLNILYC

VKIANFRTLVFRLLRQNISPKTPRLLGACVLISAFTTLLHVVLRETLHLSNFVSKRNGTM

FGTNEDILSLVISPVLSSFVQLTINVTCASLLIRSLRRHIKKMHRNATSLWSPQTEAHIG

AMKLMIYFLVLYIPYSLTSLICYLPYVKVDLRLRCVCIILSTLYYPGHSVLIIFTH

> L_timidus_T2R7

LAISRFWLLCVILLDCFMLVLYPDVYATGKEMRIIDFFWTLTNHLNVWFATCLSIFYFLK

IANFFHPLFLWMKWRIDRMIPGILLGCVALSVFISLPVTENLNDDFRRCVKTKRKTNITL

RCRVQKAGYASTKIYLNLLTLLPFSASLISFLLLILSLWRHTRKMQLNSTGYRDPSTEAH

MGAMKAVISFLFLFVTYYLAFLIATSSYFLPETELAVIWGELIALIYPSSHSFILILGNK

KL

> L_timidus_T2R9

RAISLVDIILMSLAISRICLLCVISADGFVMALFPDIHDSGELTRILDAVWTLANNLSVW

FTCCLSIFYLLKIANISHPCFLWLKLKINKVILGILLGSSLVSLVISVSMNDDMWCQFLK

TIHEGNITCQFKVSKIPNALKQITSNLGAMVPFTLCLISFFLLLSSLFRHTKQMKLHATG

TRDPSTQVHMKAIKAVVIFLLLLILYYAVFLVMTSSLLIPQGKLVVMIGGVIALIFPTSH

SFILIMGNSKLREAFLXXLRSVKVFHK

> L_timidus_T2R16

QRLSPVDMILISLGICRFCLQWISMLCDFFTYFKPNCTYLALTLPWEFINVLTFWLTSLL

AVYYCVKISSFTHPIFLWLRWRIPRLVPWLLLGSVMITCVTIIPSAIRTYIRIRLITLQQ

LPRNNTEIEKLHVFEQYLTIPHKLIALSVPFLLFLAAIILLMVSLAQHREQMQHCDXGGD

NSRMKAHVTALRSLSIFFIFFSSYFLALFITFLGTILDRRSWFWFWEAVIYAIVCIHSI

> L_europaeus_T2R1

SCLAVSRICLQLLLFYMNLFFLSLTQVSTFVENFVIVLFTNEVGLWLATWLGVFYCIKIT

NFHHPLFLWLKMRISRLLPWLVLGSLLCAAISSGICSMQSWMSLEKLLQTFLSKNATNPI

KEISALSSFSVIGGFPFPFFIFLVAALLLILSLGRHTWQLRSSTAGTRFPRRSAHVRALL

SVLSFLFLYFSHFVVVLLLSSQVFPPGSLTFMLCLLISGAYPSGHSVILILGNSKLKQNA

K

> L_europaeus_T2R4

PIQLGHHQTSNARGLFLLNIIYHTAAGVERSVYISLFLQVCWMFLDSISVWFVTLLNILY

CVKIANFRTLVFRLLRQNISPKTPRLLGACVLISAFTTLLHVVLRETLPVSNFVSKRNGT

MFGTNEDILSLVISPVLSSFVQLTINVTCASLLIHSLRRHIKKMGRNATSLWSPQTEAHI

GAMKLMIYFLVLYIPYSLTSLICYLPYVKVDLRLRCVCIILSTLYYPGHSVLIIFTHPKL

K

> L_europaeus_T2R7

LAISRFWLLCVILLDCFMLVLYPDVYATGKEMRIIDFFWTLTNHLNVWFATCLSIFYFLK

IANFFHPLFLWMKWRIDRMIPGILLGCVALSVFISLPVTENLNDDFRRCVKTKRKTNITL

RCRVQKAGYASTKIYLNLLTLLPFSASLISFLLLILSLWRHTRKMQLNSTGYRDPSTEAH

MGAMKAVISFLFLFVTYYLAFLIATSSYFLPETELAVIWGELVALIYPSSHSFILIL

> L_europaeus_T2R9

YKRAISLVDIILMSLAISRICLLCVISADGFVMALFPDIHDSGELTRILDAVWTLANNLS

VWFTCCLSIFYLLKIANISHPCFLWLKLKINKVILGILLGSSLVSLVISVSMNDDMWCQF

LKTIHEGNITCQFKVSKIPNALKQITSNLGAMVPFTLCLISFFLLLSSLFRHTKQMKLHA

TGTRDASTQVHMKAIKAVVIFLLLLILYYAIFLVMTSSLLIPQGKLVVMIGGVIALIFPS

SHSFILIMGNSKLREAFLXVLXSVKV

> L_europaeus_T2R16

SXRLSPVDMILISLGICRFCLQWISMLCDFFIYFKPNRTYLALTLPWEFINILTFWLTSL

LAVYYCVKISSFTHPIFLWLRWRIPRLVPWLLLGSVMITCVTIIPSAIRTYIRIRLITLQ

QLPRNNTEIEKLHVLEQYLTIPHKLVALSVPFLLFLAAIILLMVSLAQHREQMQHCDTGG

DNSRMKAHVTALRSLSIFFIFFSSYFLALFITFLGTILDRRSWFWFWEAVIYAIVCIHSI

S

> O_cuniculus_algirus_T2R1

SCLAVSRICLQLLFFFMNLFFLSLIQVSTSLESFVIVLFTNEVGLWLATWLGVFYCIKIT

TFHHPLFLWLKMRISRLLPWLVLGSLLCAAISSGTCSTESWMSFQKALQTFFSRNATSPI

KETSALSSFFVFGGFPFPFLVFLVAALLLILSLGRHTWQLRSPTAGTRFPSRSAHVRALL

SVLSFLFLYFSHLVVALLLSSQVFPPGSLMFMLCLLISGAYPSGHSVILILGNSKLKQNA

KRLL

> O_cuniculus_algirus_T2R3

VGHSSNWVKSKSISLSDFLITNLAFSRVILLWIFLTDGLLLVFSYGTHDSGIMMKVIDIF

WTFTNHLSIWLATCLGVLYCLKIASFSHTAFLWLKWRVSRVVVWMLLGALLFAGSSTVSL

VNEFKIHSVLEEAGDSENATELTRKKKGEYELMHVLMALWYSPPLILSLASYVLLILSLG

RHTRQMQQNGVSPRDQSTEAHKKAIRIILSFLFLFLLYFLAFLTLSSSDFLPGTXVVKMI

GEVITMSCPTVHSCILILGKRTS.SRHLWG

> O_cuniculus_algirus_T2R4

TVVSYKSWAKSSRNSSSDRILFSLGITRFLTLGLFLLNIIYHTVANVERSVYVSIFFLAC

LMFLDSVSVWFVTLLNILYCVKIANFQTSVFRLLKRNISPTTPRLLGACVLVSALTTVLH

VVFRETLPFSNFVSRRNGTMFGTNEDISSLAISSVLSSFVQLTINVTCASLLIHSLRRHI

KKMQGNATGLWSPQTEAHVGAMKLMICFLVLYIPYSVSSLICFLPYVKVDSRIRCVCIIL

STLYHP

> O_cuniculus_algirus_T2R7

SLAISRFWLLCVILLDCFILVLYPDVYATGKEMRIIDFFWTLTNHLNVWFATCLSIFYFL

KIANFFHPLFLWMKWRIDRMIPGILLGCVALSVFISLPVTENLNDDFRRCVKTKKKTNIT

LRCRVQKAGYASTKIYLNLLTLLPFSASLISFLLLILSLWRHTRKMQLNSTGCRDPSTEA

HMGAMKAVISFLFLFVAYYLAFLIATSSYFLPETELAVIWGELIALIYPSSHSFILIL

> O_cuniculus_algirus_T2R9

RVISLVDIILMSLAISRICLLCVISVDGFVMVLFPDIYDSGELSGILDAFWSLANNLSVW

FSSCLSIFYLLKIANISHPCFLWLKLKINKVIPGILLGSSLVSLVISVSMNDDMWCQFLK

TIHEENITCEFKVSKIPNTFKQITSNLGATVPFILCLISFFLLLFSLFRHTKRMKLHATG

TRDPSTQVHMKAIKAVVIFLLLLILYYAVFLVITSNFLIPQGKLVVMIGGVISLIFPSSH

SFILIMGNSKLKGSFPEGVKVCEGFPQ

> O_cuniculus_algirus_T2R16

LSPVDMILTSLGVCRFCQQWISMLCDFFFYFKPNCVYLTLTLTWEFINVLTFWLTSLLAV

YYCVKVSSFTHPIFLWLRWRIPRLVPWLLLGSVMITCVTIIPSAIRTYIRVRLITLQQLP

RNNTEIEKLYVFEQYLTTPHKLIALSVPFLLFLAAIILLMVSLAQHREQMQHRDTGGGNS

RMKAHVTALKSLSIFFIFFSSYFLALLITFLGTIVDRRSWFWLW

> O_cuniculus_algirus_T2R41

ASSDTILASLSAFRICLQCVGLGDTFFSYLHQEEYYRGRAHEFLHLHWHFLNTATFWFGT

WLSVLFCLKIANFSHPAFLWLKWRLPRLVPQFLLGFALLSFITTLLLFWGNHVLHQGLIL

RKFSGNTTYKEWKRRMEFHFFLPVKLVTLSIPCCFFLLSLVLLISSLRRHAWRMKRNAHS

LQDSSGQAHNRALRSLVSFLTLYTLSFTSLVINGTFFSSLESEWYWPWQIILYLCICLHP

FILISSNLKLRSVF

> O_cuniculus_cuniculus_T2R1

SCLAVSRICLQLLFFFMNLFFLSLIQVSTSLESFVIVLFTNEVGLWLATWLGVFYCIKIA

TFHHPLFLWLKMRISRLLPWLVLGSLLCAAISSGTCSTESWMSFQKALQTFFSRNATSPI

KETSALSSFFVFSCFPFPFLVFLVAALLLILSLGRHTWQLRSPTAGARFPSRSAHVRALL

SVLSFLFLYFSHLVVALLLSSQVFPPGSLMFMLCLLISGAYPSGHSVILILGNSKLKQNA

KRLL

> O_cuniculus_cuniculus_T2R3

VGHSSKWVKSKSISLSDFLITNLAFSRVILLWIFLTDGLLLVFSYGTHDSGIMMKVIDIF

WTFTNHLSIWLATCLGVLYCLKIASFSHAAFLWLKWRVSRVVVWMLLGALLFAGSSTVSL

VNEFKIHSVLEEAGDSENATELTRKKKGEYELMHVLMALWYSPPLILSLASYVLLILSLG

RHTRQMQQNGVSPRDQSTEAHKKAIRIILSFLFLFLLYFLAFLTLSSSDFLPGTKVVKMI

GEVITMSCPTVHSCILILGNDKLXXTFVGD

> O_cuniculus_cuniculus_T2R4

TVVSYKSWAKSNRNSSSDRILFSLGITRFLTLGLFLLNIIYHTVANVERSVYVSIFLLAC

LMFLDSVSVWFVTLLNILYCVKIANFQTSVFRLLKRNISPTTPRLLGACVLVSAFTTVLH

VVLRETLPFSNFVSRRNGTMFGTSEDISSLVISSVLNSFVQLTINVTCASLLIHSLRRHI

KKMQGNATGLWSPQTEAHVGAMKLMICFLVLYIPYSVTSLICFLPYVKVDLRIRCVCIIL

STLYHP

> O_cuniculus_cuniculus_T2R7

SLAISRFWLLCVILLDCFILVLYPDVYATGKEMRIIDFFWTLTNHLNVWFAACLSIFYFL

KIANFFHPLFLWMKWRIDRMIPGILLGCVALSVFISLPVTENLNDDFRRCVKTKKKTNIT

LRCRVQKAGYASTKIYLNLLTLLPFSASLISFLLLILSLWRHTRKMQLNSTGCRDPSTEA

HMGAMKAVISFLFLFVAYYLAFLIATSSYFLPETELAVIWGELIALIYPSSHSFILILG

> O_cuniculus_cuniculus_T2R9

RVISLVDIILMSLAISRICLLCVISVDGFVMVLFPDIYDSGELSGILDAFWSLANNLSVW

FSSCLSIFYLLKIANISHPCFLWLKLKINKVIPGILLGSSLVSLVISVSMNDDMWCQFLK

TIHEENITCEFKVSKIPNTFKQITSNLGATVPFILCLISFFLLLFSLFRHTKRMKLHATG

TRDPSTQVHMKAIKAVVIFLLLLILYYAVFLVITSNFLIPQGKLVVMIGGVISLIFPSSH

SFILIMGNSKLKEAFLXVLKVCEGFPQ

> O_cuniculus_cuniculus_T2R16

LSPVDMILTSLGVCRFCLQWISMLCDFFFYFKPNCVYLTLTLTWEFINVLTFWLTSLLAV

YYCVKVSSFTHPIFLWLRWRIPRLVPWLLLGSVMITCVTIIPSAIRTYIRVRLITLQQLP

RNNTEIEKLHVFEQYLTTPHKLIALSVPFLLFLAAIILLMVSLAQHREQMQHRDTGGGNS

RMKAHVTALKSLSIFFIFFSSYFLALLITFLGTIVDRRSWFWLWEDVIYAIVCIHSISLA

LSSPTLK

> O_cuniculus_cuniculus_T2R41

ASSDTILASLSAFRICLQCVGLGDTFFSYLHQEEYYRGRAHEFLHLHWHFLNTATFWFGT

WLSVLFCLKIANFSHPAFLWLKWRLPRLVPQFLLGFALLSFITTLLLFWGNHVLHQGLIL

RKFSGNTTYKEWKRRMEFHFFLPVKLVTLSIPCCFFLLSLVLLISSLRRHAWRMKRNAHS

LQDSSGQAHNRALRSLVSFLTLYTLSFTSLVINGTFFSSLESEWYWPWQIILYLCICLHP

F

> R_diazi_T2R7

FWLLCVILLDCFILVLYPDVYATGKEMRIIDFFWTLTNHLNVWFATCLSIFYFLKIANFF

HPLFLWLKWRIDRMIPGILLGCVALSVFISLPVTENLNDDFRRCVKTKRKTNITLRCRVQ

KAGYASTKIYLNLWTLLPFSASLISFLLLILSLWRHTRKMQLNSTGYRDPSTEAHMGAMK

AVISFLFLFVTYYLAFLIATSSYFLPETELAVIWGELIALIYPSSHSFILILGNKKLRQ

> R_diazi_T2R16

SQRLSPVDMILISLGICRFCLQWVSMLSDFFKYFKPNCTYFILTLSWEFINILTFWLTSL

LAVYYCVKISSFTHPIFLWLRWRIPRLVPWLLLGSVMIACVTIIPSAIWTYIRIRLVTLQ

HLPRNNTEIEKLHVFERYLAIPHKLTALSIPFFLFLAAIILLMVSLAQHQKQMQHHDIGG

GNSRMKAHVTALRSLSIFFIFFSSYFLALFITFLXTIVDRRSWFWLWETVIYAIVCIHSN

SLALSS

> R_diazi_T2R41

LHLHWHFLNTATFWFGTWLSVLFCLKIANFSHPAFLWLKWRLPRLVPQFLLGFALLSFIT

TLLLFWGNHVLHQGLILRKFSGNTTYKEWKRRMEFHFFLPVKLVTLSIPCCFFLLSLVLL

ISSLRRHAWRMKRNAHSLQDSSGQAHNRALRSLVSFLTLYTLSFTSLVINGTFFSSLESE

WYWPWQIILYLCICLHPFILISSNLKLRSVFRRLL

> S_floridanus_T2R4

LGITRFLTLGLFLLNXXYHTAPNAERSVYIAIFFLVCWMFLDSVTVWFVTLLNILYCVKI

TNFQPSVFRLLKRNISPKTPRLLGACVLISVFTTLLHVVLRETLPFSSFVSKRNGTIFGT

NEDISSLVTSSVLSSFVQLTINVTCASLLIHSLRRHIKKMQRNATGLWSPQTEAHIGAMK

LMIYFLVLYIPYSLTTLICYLPYVKVDLRIRCVCIILSTLYHPGHSVSHHFHSS.AK

> S_floridanus_T2R16

RLSPVDMILISLGICRFCQQWISMLFDFFNYFKPNCRYFTLALSWEFINILTFWLTSLLA

VYYCVKISSFTHAIFLWLRWRIPRLVPWLLLGSVMITCVTIIPSAVWTYIRIRLINLQHL

PRNNTEIEKLHAFQRYLAIPHKLTALSIPFFLFLAAIILLMVSLVQHREHMQHHDTGGGN

SRMKAHVTALRSLSIFFIFFSSYFLALFITFLGTIVDRSSWFWLWEAVIYAIVCIHSISL

ALSSPTLK

> S_floridanus_T2R41

EEYYRGRAHEFLHLHWHFLNTATFWFGTWLSVLFCLKIANFSHPAFLWLKWRLPRLVPQF

LLGFALLSFITTLLLFWGNHVLHQGLILRKFSGNTTYKEWKRRMEFHFFLPVKLVTLSIP

CCFFLLSLVLLISSLRRHAWRMKRNAHSLQDSSGQAHNRALRSLVSFLTLYTLSFTSLVI

NGTFFSSLESEWYWPWQIILYLCICLHPFILISSNLKLRSVFRRLL

> O_princeps_T2R4

MILFSLGITRFLMLGLFLLNMGYYIAAKVERSVYISIFLLVCWMFFEYTSLWFVTLLNVL

YCVKISSFQPTMFLLLKRNLSLKTPWFLGACVLISAFTTLLHVMLRQTLVSSTLRTEKND

TSFDTNEDIVFLVVSSVLSSFVQFIINVTCASLLIHSLRRHIKKMQRNTTGLWNPQTEAH

VGAMKLMVYFLVLYIPHSVASLFYFLPPSVKMNWVTKAICTVISTLYPPGHSVLIIFTHR

KLKTKAKKLLCCNK

> O_princeps_T2R7

MNILMLIAAGEFSVGILGNIFIGLVNFMDWIKSRKIASIDLILTSLAISRICLLCVILLD

CFILVLYPDIYAAGKQMRIIDFFWTLTNHLNVWFATCLSIFYFLKIANFFHPVFLWMKWR

IDKLILRILLGCVALSVFISLPVTVNLNDDFRRCVKKKKMNVTLKCRVNKAGYASTKVFL

NLLTLLPFSASLISFLLLILSLWRHTRNMQLNSTGCRDPSTEAHVGAMKAVISFLVLFVA

YYLSFLIATSSYFMPETELAVILGELIALIYPSSHSFILILGNNKLRQATLSMLRKVKCT

LQWRNCQQK

> O_princeps_T2R9

MLRTTEALYLILMAGELTLGIWGNGFIVLVNCVDWLTKRSISLVDIILMSLAISRIGLLC

AVSIDGFIMVLSPEIYGNVELTGILDAFWTMANNLSVWSTCCLSIFYLLKIANISHPCFL

WMKLRTNSMILGILLGSSLISLIISVSINDDMWCNLLKITHEENITYPFKVTKIPNALKQ

ITSNLAVTI

> O_princeps_T2R41

MQPALTLSFMLLFVLLSGLGILGNSFIVLVLSREWLRHGRLLPSDKILISLSASRCCLQC

VGLGDNFYSYFYQEKYFRGVAHKFFHLYWHFMSVAAFWFGTWLSVLFCVKIANFSHPTFL

WLKWRLPRLVAWFLLGFVLMAFITTLLLFWGNHILHQGLILRKYSGNITYKEWRRKIIFH

FLLPVKLVSLSTPFSVFLLSILLLISSLRRHTCRMKLNGHSQQDSSSQAHSRALKSLLSF

LALYILSFMALIIDDTFFFSLESEWYWLWQIILYLCIFFHPFILISSNLKLRNVIRHLLS

LARGFWVAQMAVLAYTEEENRTMAHLRGNAFIGS
